# Efficacy of Nonthermal Atmospheric Pressure Plasma for Tooth Bleaching

**DOI:** 10.1155/2015/581731

**Published:** 2015-01-20

**Authors:** Seoul Hee Nam, Hae June Lee, Jin Woo Hong, Gyoo Cheon Kim

**Affiliations:** ^1^Department of Oral Anatomy, School of Dentistry, Pusan National University, Yangsan 626-870, Republic of Korea; ^2^Department of Electrical Engineering, Pusan National University, Busan 609-735, Republic of Korea; ^3^Department of Internal Medicine, School of Korean Medicine, Pusan National University, Yangsan 626-870, Republic of Korea

## Abstract

The conventional light source used for tooth bleaching has the potential to cause thermal damage, and the actual role of the light source is doubtful. In this study, we evaluated bleaching efficacy, temperature, and morphological safety after tooth bleaching with nonthermal atmospheric pressure plasma. Tooth bleaching combined with plasma had improved efficacy in providing a higher level of brightness. The temperature of the pulp chamber was maintained around 37°C, indicating that the plasma does not cause any thermal damage. The morphological results of tooth bleaching with plasma did not affect mineral composition under scanning electron microscopy (SEM) observations. On the basis of these results, the application of plasma and low concentration of 15% carbamide peroxide (CP) has a high capability for effective tooth bleaching. It can be documented that plasma is a safe energe source, which has no deleterious effects on the tooth surface.

## 1. Introduction

The demand for an improved appearance and a whiter smile has made tooth bleaching a very popular dental procedure [1]. As tooth bleaching continues to grow in popularity, numerous patients seek dental assistance for cosmetic improvement of tooth discoloration.

Clinical bleaching agents use a high concentration of hydrogen peroxide (HP; H_2_O_2_) or carbamide peroxide (CP; CH_6_N_2_O_3_), both of which provide oxidative power [2]. They diffuse into and through enamel to reach dentin where they react with the organic chromogens responsible for the major color factors of teeth [3]. Most of the current in-office tooth bleaching techniques use a light source or heat as the activator to improve bleaching efficacy by accelerating the free radical (HO_2_^∙^, HO^∙^) dissociation rate [4]. These free radicals have low molecular weights and are able to denature proteins, penetrate enamel, and diffuse through the organic matrix of dentin to exert their bleaching effect [5], thus reducing the time needed to whiten teeth [6].

However, tooth bleaching with a high concentration of HP bleaching agent and a high-intensity light source has the potential to cause adverse effects on the surface enamel [7, 8]. Consequently, the bleached surfaces will incur greater or faster morphological and structural changes in enamel [9, 10]. Furthermore, studies have demonstrated that the heat generated by the light sources used in tooth bleaching may also increase the temperature at the tooth surface and inside the pulp chamber [11, 12]. It is commonly believed that such temperature increases associated with certain tooth bleaching procedures pose a serious threat to pulp vitality [13, 14].

Plasma is the fourth state of matter, the others being solid, liquid, and gas. Plasma forms when gas is ionized. It has the ability to conduct electricity and respond to electromagnetic fields, because plasma contains many radicals, a strong electric field, and charged particles. Since 2000, nonthermal atmospheric pressure plasma sources have been used for biomedical applications such as bacterial sterilization, cancer treatment, wound healing, and dental applications [15–18]. If plasma provides maximum efficiency in bleaching and safety, it can be widely used in dental clinics as a novel technique. Therefore, the aim of the present study was to demonstrate the bleaching efficacy and morphological effect of plasma with a low concentration of 15% CP.

## 2. Materials and Methods

### 2.1. Plasma Device

**Figure 1:**
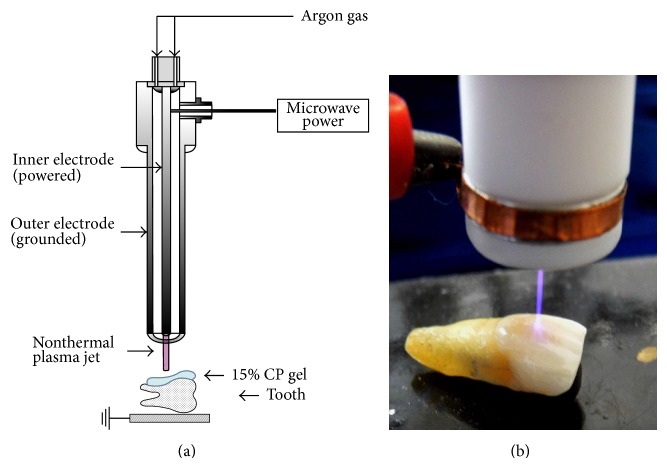
The application of nonthermal atmospheric pressure plasma for tooth bleaching with low concentrations of 15% CP. (a) The plasma device and (b) the process during bleaching treatment.

Figures 1(a) and 1(b) show a schematic of the nonthermal atmospheric pressure plasma and a photo of tooth bleaching using a microwave-driven plasma source. It was operated by a palm-sized power module with a net 2.5 W of input power. A low temperature plasma jet was driven by argon (Ar) gas with a flow rate of 2.5 L/min^−1^ in air. The excited Ar species and/or energetic electrons from the plasma can make collisions with the ions, charged particles. The jet length extends up to 3 cm beyond the end of plasma device and the plasma jet can be touched without discomfort. The plasma diffused up to a few centimeters into the surrounding atmosphere to reach the tooth surface. A detailed description of the electrical and physical features of the plasma jet has reported this previously [16, 18].

### 2.2. Tooth Preparation

Twenty freshly extracted intact human teeth were stored in 0.4% sodium azide solution at room temperature until required. Any teeth with signs of fracture, dental caries, or structural anomalies were discarded. Before experimental use, all teeth were removed with a soft-tissue ultrasonic scaler and polished in a dental rubber cup with water or pumice slurry prophylaxis. The roots were cut with water-cooling using a diamond saw (Struers Minitom, Copenhagen, Denmark) approximately 0.5 mm below the cement-enamel junction. The crown was cut in half longitudinally and cut surfaces were coated with two layers of nail varnish. The study was approved by the Research Ethics Committee of the Pusan National University Yangsan Hospital, Yangsan, Republic of Korea (Approval number 04-2012-010).

### 2.3. Tooth Bleaching Procedure

Before the tooth bleaching, the buccal surface of each tooth was photographed using a digital-imaging system provided by a stereomicroscope (SZ-CTV, Olympus, Tokyo, Japan) at 10x magnification. The teeth were randomly assigned to two groups (*n* = 10). Group 1 received a 15% CP gel application which contained 5.4% HP (every 10 min, Kool White 15%, Pac-Dent International, Walnut, CA, USA) and plasma for 30 min. The tip of the static plasma device was positioned at a 1 cm distance from the tooth surface. These bleaching procedures were repeated three times during 30 min treatment. Group 2 was treated with a uniform 1 mm layer of 15% CP alone without plasma application. After 10 min application, the teeth were thoroughly rinsed to remove all the gel with distilled water and gently dried with sterile gauze. The procedure was repeated at 10 min intervals so that the total bleaching time was 30 min.

### 2.4. Analysis of the Bleaching Efficacy

The tooth images were captured with a 10x magnification digital-imaging system consisting of a stereomicroscope connected to a camera (Pixel Link PL-B686 CU, Canada) at 10 min intervals. The images were loaded onto a computer through Image-Pro Plus 5.1 software (Media Cybernetics Inc., Washington, DC, USA). The overall color changes (Δ*E*) were assessed on the basis of the Commission Internationale de L'Eclairage (CIE, 1979) Lab Color System [19]. The *L* ^*^ value represents the degree of lightness from 0 (black) to 100 (white). The *a* ^*^ value represents the degree of greenness (negative *a* ^*^) or redness (positive *a* ^*^), while the *b* ^*^ value represents the degree of blueness (negative *b* ^*^) or yellowness (positive *b* ^*^). The differences in Δ*E* values were calculated to compare the color changes and are expressed by the formula

(1) $$\begin{array}{c} \Delta E=\sqrt{{\left(\Delta L^{*}\right)}^{2}+{\left(\Delta a^{*}\right)}^{2}+{\left(\Delta b^{*}\right)}^{2}}. \end{array}$$

### 2.5. Measurement of Tooth Temperature

The temperature increase in each tooth was measured independently from the tooth bleaching experiments using a fiber-optic temperature measurement system (FTI-10 fiber-optic signal conditioner, FOT-L-SD fiber-optic temperature sensor; FISO Technologies Inc., Quebec, Canada). The fiber-optic temperature sensor was located within the pulp chamber. The distance between the emitting tip of the plasma source and the fiber-optic temperature sensor was set at 1 cm during “plasma-on” for 30 min at room temperature (25°C).

### 2.6. Surface Morphology

Immediately after drying, the images were then assessed using scanning electron microscopy (SEM; S3500N, Hitachi Co., Tokyo, Japan) at 15 kV accelerating voltage. The images were then assessed at 2,000x magnification.

### 2.7. Statistical Analysis

Statistical analysis was performed with the SPSS computer program (SPSS Inc., Version 18.0, Chicago, IL, USA). Student's *t*-test was used to determine the difference in Δ*E* values between tooth bleaching with plasma and that without plasma. The level of significance was defined at *P* < 0.05.

## 3. Results and Discussion

### 3.1. Bleaching Efficacy

Today's bleaching systems are the result of efforts to increase patient benefits in terms of better bleaching efficacy and ease of use [20]. Various light sources are being used in dental clinics, as they are known to reduce the total treatment time; however, the bleaching effects caused by such light sources remain unclear [21]. This study was conducted using nonthermal atmospheric pressure plasma as a light source to solve several problems associated with current tooth bleaching methods. Tooth bleaching efficacy using plasma plus 15% CP significantly increased during the 30 min treatment. Plasma produces a larger amount of hydroxyl radicals (^∙^OH) [16] which play an important role in tooth bleaching. Hence, there is synergistic enhancement resulting in a more rapid bleaching efficacy. Nam et al. [22] have reported that plasma application resulted in more improved tooth bleaching than in conventional methods. In this study, Table 1 reveals that there was a statistically significant difference in the bleaching efficacy between Group 1 and Group 2 (*P* < 0.05). The mean Δ*E* ± standard deviation (SD) after 30 min bleaching was 29.91 ± 1.74 (Group 1) and 8.30 ± 1.15 (Group 2). Group 1A was 3.7 times brighter than Group 1B. The teeth treated with plasma showed higher Δ*E* values than those without plasma. Remarkable color change occurred over time in the group treated with plasma. These results suggest that the amount of ^∙^OH generated from plasma diffuses quickly through the surface of the enamel and breaks down the conjugated bonds in protein chains (stains) into a single bond, which increases bleaching efficacy.

### 3.2. Temperature Measurement

**Figure 2:**
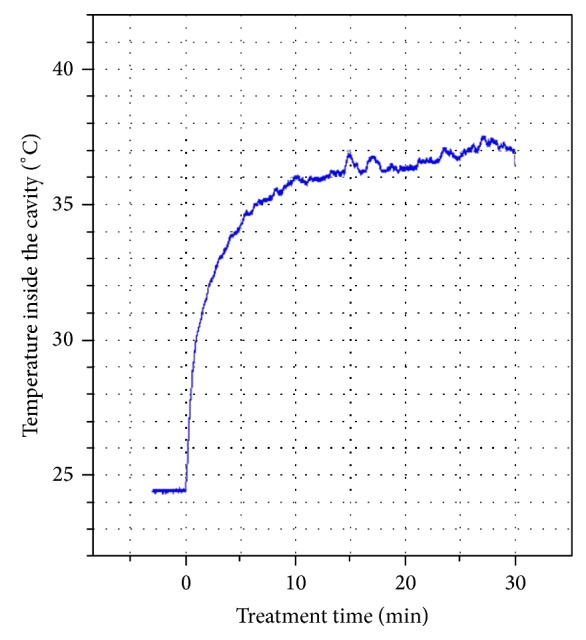
Measurement of the dental cavity temperature during the bleaching treatment.

Many studies have demonstrated thermal damage induced by light sources. It is also known that temperature increases in the pulp chamber may be a serious threat to the vitality of the pulp [23]. Eriksson et al. [24] showed that pulp temperatures in excess of 42.5°C may induce irreversible pulpitis. In conventional bleaching procedures, temperatures can easily exceed these values [22]. However, the temperature of a tooth treated with plasma did not increase above body temperature. The plasma took about 10 min to reach a steady-state temperature, and thereafter the temperature was continuously maintained around 37°C throughout the bleaching duration (Figure 2). Due to the fact that the temperature of plasma treatment is similar to human body temperature, therefore, it can be concluded that plasma maintains a low and consistent pulp temperature during tooth bleaching and avoids undue risk to the pulp.

### 3.3. Tooth Surface Evaluation

**Figure 3:**
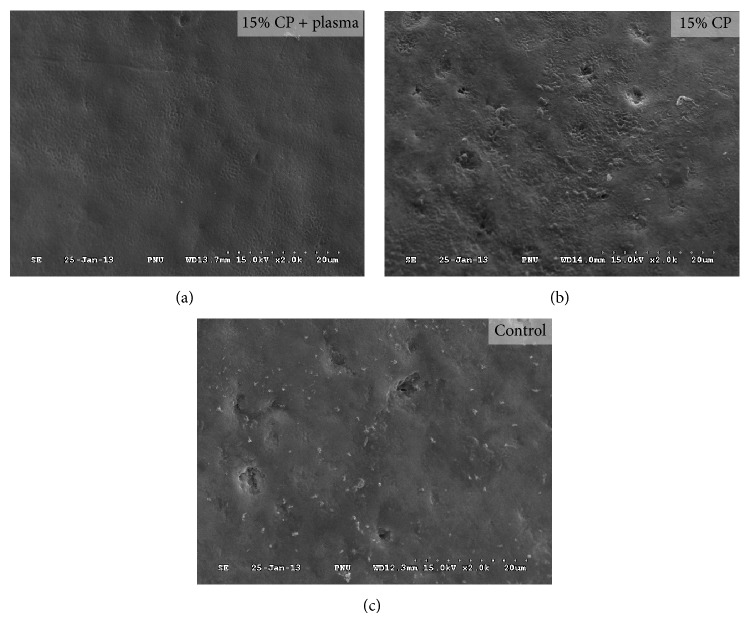
SEM photomicrographs of enamel surface morphology for each group at 2000x magnification.

In most cases, SEM studies were used to analyze the morphological changes of the tooth surface at high resolution and magnification [25]. According to a study by Fu et al. [26], bleached enamel surfaces resulted in morphological changes to the tooth surface.  Figure 3 shows SEM photomicrographs of tooth enamel surfaces bleached with or without plasma. After combining plasma with a low concentration of 15% CP, the bleached enamel surface was observed to be smooth. On the other hand, the enamel surface treated with 15% CP alone results in a much rougher surface than the control. The results of this study show that tooth bleaching with plasma does not cause morphological changes to tooth enamel. The tooth surface, in fact, remains smooth. This could explain how plasma effectively removes colored proteins [16, 27]. Plasma application for tooth bleaching does not dissolve the mineral content of tooth enamel, indicating that plasma did not influence the demineralization effects of bleached teeth. Based on the results of this study, the application of plasma for tooth bleaching quickly decomposed HP to ^∙^OH radical; thereby it did not cause the deleterious effects on enamel surface.

## 4. Conclusions

The combination of nonthermal atmospheric pressure plasma with 15% CP has a greater capability for effective tooth bleaching than conventional light sources. The tooth surface temperature was maintained around 37°C, indicating that the plasma does not cause any thermal damage to the tooth. The application of plasma did not cause any structural changes to the bleached surface. Nonthermal atmospheric pressure plasma has been proven to have no deleterious effect on bleached enamel. Therefore, tooth bleaching techniques using plasma represent a method of cosmetic dentistry with many practical applications in the future.

## Figures and Tables

**Table 1:** The mean Δ*E* ± SD after tooth bleaching with and without plasma and P values using Student's t-test.

Observation times	Bleaching agents	*N*	Mean Δ*E* ± SD		*t*-test *P* values
			Plasma	Without plasma	
10 min	15% CP	20	17.82 ± 1.74	5.87 ± 0.81	0.000^*^
20 min	15% CP	20	23.48 ± 2.30	6.98 ± 0.93	0.000^*^
30 min	15% CP	20	29.91 ± 1.74	8.30 ± 1.15	0.000^*^

CP: carbamide peroxide, Δ*E*: overall color changes, and SD: standard deviation.

^*^Δ*E* values show significant differences between the two groups (*P* < 0.05).

## Abbreviations

SEM: Scanning electron microscopy
CP: Carbamide peroxide.
